# Combined effects of psychological and life style factors on pain intensity and/or disability in patients with chronic low back pain: A cross-sectional study

**DOI:** 10.1192/j.eurpsy.2024.191

**Published:** 2024-08-27

**Authors:** E. Tsatsaraki, I. Bouloukaki, G. Kontakis, A. Vakis, A. Miliaras, M. Basta

**Affiliations:** ^1^Surgery; ^2^Social and Family Medicine; ^3^Orthopaedics and Traumatology; ^4^Neurosurgery; ^5^Psychiatry, University of Crete, School of Medicine, Herkalion, Greece

## Abstract

**Introduction:**

Chronic Lower Back Pain (CLBP) is a frequently encountered health issue in primary care settings, leading to global disability and imposing a considerable economic burden.

**Objectives:**

This study aimed to: (1) compare socio-demographic, health, lifestyle (sleep, physical activity) and psychological factors (depression, anxiety) between people with and without CLBP; and (2) quantify the correlations between these psychological and lifestyle factors, and clinical outcomes (intensity of CLBP and CLBP-related disability) in people with CLBP after considering other confounders.

**Methods:**

A cross-sectional study was undertaken at the neurosurgery and orthopedic outpatient department of Heraklion University Hospital between 2019-2021. Two hundred fifty three volunteers with CLBP and 116 without CLBP provided sociodemographic information, daily habits, medical history, subjective sleep/ sleep complaints, low back pain intensity and disability using a10-point numeric Visual Analogue Scale pain rating scale and Quebec Back Pain Disability Scale, as well as questions assessing impact of pain on mobility, self-care, routine activities and psychological status, respectively. Participants also completed the Zung Self-Rating Scale (SDS) for self-assessment of depression and Self-rating anxiety scale (SAS). Associations among CLBP, demographics, psychosocial or sleep disorders parameters and clinical outcomes were analyzed using multivariate models.

**Results:**

People with CLBP exhibited a substantially greater prevalence of depressive, insomnia and obstructive sleep apnea (OSA) symptoms than controls (*p* < 0.05). CLBP diagnosis was independently correlated with female gender, older age, as well as worse physical and mental health outcomes measured by (i) higher level of sleep symptoms such as sleepiness, OSA and insomnia symptoms and (ii) higher prevalence of physician-diagnosed depression, and moderate to severe depressive symptoms. The level of functional disability for CBLP patients (based on Quebec score) was independently associated with age, physician diagnosed depression, lower educational status, moderate to severe depressive symptoms and OSA symptoms. The combination of moderate to severe depressive symptoms with OSA or insomnia symptoms was the most important predictive factor for functional disability for CBLP patients (OR 13.686, 95% CI 4.581-40.885; p<0.001).

**Conclusions:**

Depressive symptoms and subjective sleep disorders appear to relate to greater CLBP-intensity and/or CLBP-related disability in people with CLBP. To achieve the desired outcomes when treating patients with chronic CLBP, it is essential to employ a holistic approach, involving assessment and management of their psychological comorbidities, and sleep issues, that may improve quality of life in these patients.

**Disclosure of Interest:**

None Declared

